# Study on articular surface morphology of atlantoaxial lateral mass based on differential manifold

**DOI:** 10.1186/s13018-023-04410-3

**Published:** 2023-12-02

**Authors:** Zeyuan Zhang, Yao Zhao, Dean Chou, Shuhao Zhang, Ruifang Zhou, Zeyu Ma, Limin Wang, Zhong Yu, Yilin Liu, Yuqiang Wang

**Affiliations:** 1 Department of the Orthopaedic Surgery, The First Affiliated Hospital of Zhengzhou University, No.1 Jianshe East Road, Zhengzhou, 450052 China; 2 Department of the Neurosurgery, Columbia University, New York, USA; 3 School of Mathematics and Information Sciences, Zhongyuan University of Technology, Zhengzhou, China

**Keywords:** Articular surface of lateral atlantoaxial mass, Craniocervical junction abnormalities, Differential manifold, Atlantoaxial fusion apparatus

## Abstract

**Objectives:**

To propose a surface reconstruction algorithm based on a differential manifold (a space with local Euclidean space properties), which can be used for processing of clinical images and for modeling of the atlantoaxial joint. To describe the ideal anatomy of the lateral atlantoaxial articular surface by measuring the anatomical data.

**Methods:**

Computed tomography data of 80 healthy subjects who underwent cervical spine examinations at our institution were collected between October 2019 and June 2022, including 46 males and 34 females, aged 37.8 ± 5.1 years (28–59 years). A differential manifold surface reconstruction algorithm was used to generate the model based on DICOM data derived by Vision PACS system. The lateral mass articular surface was measured and compared in terms of its sagittal diameter, transverse diameter, articular surface area, articular curvature and joint space height.

**Results:**

There was no statistically significant difference between left and right sides of the measured data in normal adults (*P* > 0.05). The atlantoaxial articular surface sagittal diameter length was (15.83 ± 1.85) and (16.22 ± 1.57) mm on average, respectively. The transverse diameter length of the articular surface was (16.29 ± 2.16) and (16.49 ± 1.84) mm. The lateral articular surface area was (166.53 ± 7.69) and (174.48 ± 6.73) mm^2^ and the curvature was (164.03 ± 5.27) and (153.23 ± 9.03)°, respectively. The joint space height was 3.05 ± 0.11mm, respectively. There is an irregular articular space in the lateral mass of atlantoaxial, and both upper and lower surfaces of the articular space are concave. A sagittal plane view shows that the inferior articular surface of the atlas is mainly concave above; however, the superior articular surface of the axis is mainly convex above. In the coronal plane, the inferior articular surface of the atlas is mostly concave above, with most concave vertices located in the medial region, and the superior articular surface of the axis is mainly concave below, with most convex vertices located centrally and laterally.

**Conclusion:**

A differential manifold algorithm can effectively process atlantoaxial imaging data, fit and control mesh topology, and reconstruct curved surfaces to meet clinical measurement applications with high accuracy and efficiency; the articular surface of the lateral mass of atlantoaxial mass in normal adults has relatively constant sagittal diameter, transverse diameter and area. The distance difference between joint spaces is small, but the shape difference of articular surfaces differs greatly.

## Introduction

Craniocervical junction abnormalities (CJA) are congenital bony anomalies of the occipital and atlantoaxial that occur in the craniocervical junction region, which is the transition between the skull and the cervical spine [1]. There is a possibility of quadriplegia in severe cases, and medulla oblongata compression may result in respiratory distress. Due to the special anatomical location of the atlantoaxial spine and the complex anatomical structures of the surrounding blood vessels and nerves, the treatment of atlantoaxial spine disorders can also be relatively difficult. With the introduction of modern imaging technologies—computer tomography (CT), magnetic resonance imaging (MRI)—there is better understanding of these abnormalities. However, the treatment of this pathology can be difficult. The goals of treatment of CJA are to restore the anatomy of the atlantoaxial spine, relieve the compression of the spinal cord, and stabilize the atlantoaxial complex [2]. A common surgical procedure to treat atlantoaxial disorders is atlantoaxial fusion, and biomechanical studies have shown that atlantoaxial screws and rods can provide excellent stability when combined with atlantoaxial lateral mass interarticular fusion devices [3, 4]. Previous studies on the atlantoaxial facet joint, which is irregular in shape and has a certain angle of inclination [5] and curvature, are less well-reported, so measurement of the anatomical data on the facet is important for the design of the atlantoaxial facet fusion device.

A manifold is a mathematical space that locally resembles Euclidean space, including curved surfaces of various latitudes, such as spheres and curved planes. This technique is mostly applied in rock mechanics and engineering, and with the popularity and development of multidisciplinary intersections, the manifold has been used in many medical imaging studies in the past decade [6]. Based on previous studies, we apply the implicit surface reconstruction algorithm of the differential manifold for the first time to process the optimized point cloud data to obtain the final atlantoaxial lateral mass articular surface model and then measure the anatomical data related to the articular surface.

In this study, medical imaging data was processed using an implicit surface algorithm with differential manifold. A model of the atlantoaxial lateral mass was reconstructed, the anatomical parameters associated with the articular surface were measured, the morphological features associated with the articular surface were analyzed, data for the design of anatomical atlantoaxial lateral mass fusion devices were analyzed, and a theoretical basis for treating CJA is provided.

## Materials and methods

### Study subjects

A retrospective analysis of clinical data was conducted at the Department of Imaging of the First Affiliated Hospital of Zhengzhou University between October 2019 and June 2022 for 80 normal adults who had 64-slice CT examinations of their cervical spines. Atlantoaxial congenital or acquired deformity, atlantoaxial trauma, atlantoaxial infectious or neoplastic lesions, and history of previous atlantoaxial surgery were excluded. There were 46 males and 34 females with ages ranging from 28 to 59 years (37.8 ± 5.1 years), heights ranging from 155–180 cm (161.54 ± 7.03 cm), and weights from 36–80 kg (58.89 ± 11.48 kg) (Table 1). No significant differences were observed when comparing general information such as gender, height, and weight. This study was approved by the Medical Ethics Committee of the First Affiliated Hospital of Zhengzhou University.

**Table 1:** Demography of the study groups ($\overline{x }$±s)

Basic information	Amount
Total	80 cases
Gender	
Male Female	46 cases 34 cases
Age (year)	37.8 ± 5.1
Height (cm)	161.54 ± 7.03
Weight (Kg)	58.89 ± 11.48

### Articular surface flow reconstruction

#### CT data collection

Scanning was performed using Philips 64-slice multilayer spiral CT. The examinee was placed in a supine position, and the neck was adjusted in neutral position. Scanning range was C1 to C7 with a focus on the skull base to C3. Scanning parameters were using tube voltage 120–135 kV, tube current 250 mA, and thin layer thickness 0.625 mm, pitch 0.4 mm. After scanning, the raw data were uploaded to the Vision PACS (picture archiving and communication system) image workstation and exported to DICOM (digital Imaging and communications in medicine) format using the “image storage” function included in the image workstation. The image was exported in DICOM format using the “image storage” function of the image workstation.

#### DICOM data preprocessing

**Fig. 1:**
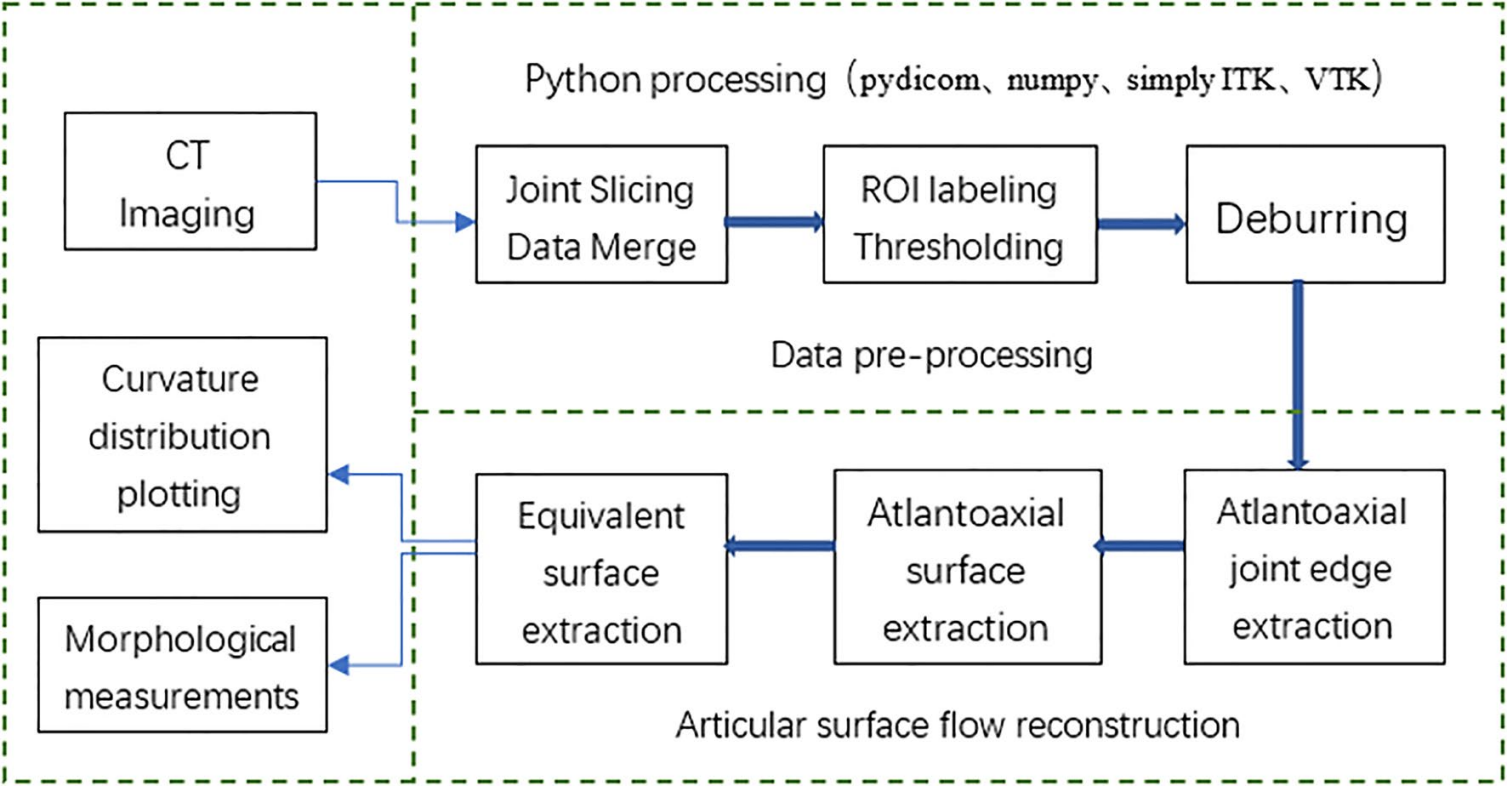
Atlantoaxial facet flow reconstruction process

A Python environment with SimpleITK (insight toolkit) package was used to open the above CT raw dicom format data, first merging the slice data, and then reading the origin and spatial resolution of the first slice. The image data in the dicom file are extracted as numpy arrays for subsequent processing. ROI labeling was performed by three experienced radiologists with brief training. The annotated ROI area is thresholded, and the skeletal threshold is set to 226 to separate the skeleton from the muscle, etc., and extract the atlantoaxial joint skeleton. The raw data of the extracted atlantoaxial joint bones are located in the space, and the deburring algorithm is designed to remove the burrs around the atlantoaxial joint, and the differential image is obtained by the check score algorithm to extract the bone edges. The size of the array extracted from the atlantoaxial joint gap is (50,55,20). Preprocess the extracted atlantoaxial bones and complete the data (Fig. 1).

### Articular surface flow reconstruction

**Fig. 2:**
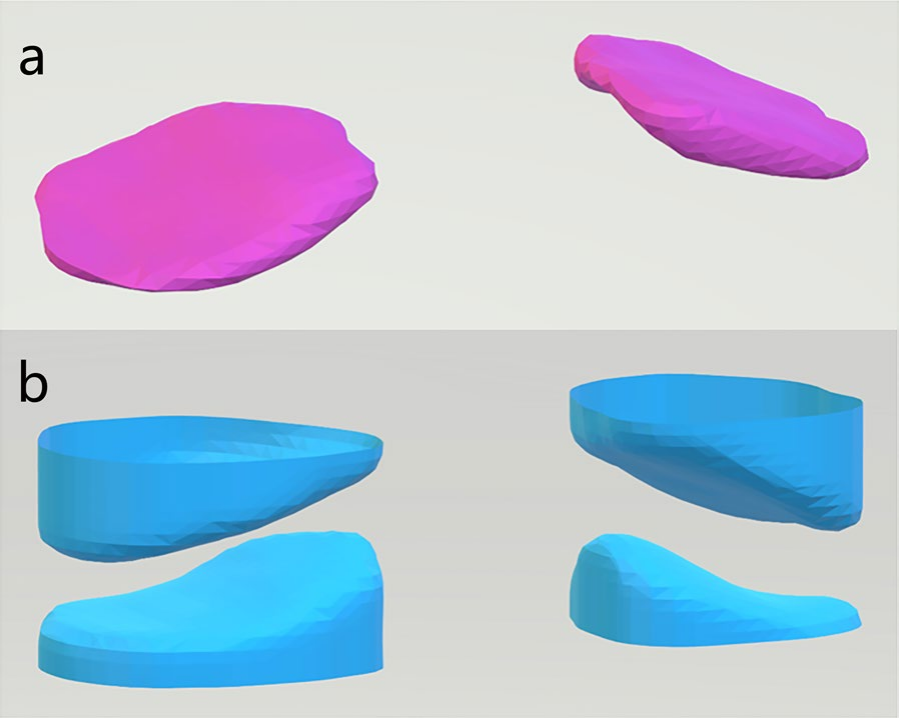
**a** Initial extraction of the atlantoaxial joint space **b** Atlantoaxial articular surface after smoothing treatment (the joint gap has been raised to facilitate observation of the joint surface)

There are two irregular and complex surfaces on the lateral mass of the atlantoaxial spine, and mathematical models cannot adequately describe their morphology. In this study, the articular surfaces of the lateral mass of the atlantoaxial spine are considered smooth surfaces in three-dimensional space (Riemann manifold), which is also a kind of differential flow shape. Each coronal section of the ROI is different, and an algorithm is designed based on the original data and the differential data to extract the skeletal gap of the atlantoaxial joint (Fig. 2a).

The extracted gap model is Gaussian filtered and smoothed to remove noise (Fig. 2b). For the smoothed atlantoaxial joint gap model, algorithms were designed to extract the upper and lower surfaces of the atlantoaxial joint. The slope, curvature and concavity were calculated for each atlantoaxial joint surface coronal section of the profile line. The point cloud model of the atlantoaxial joint surface is subsequently processed using vtk (visualization toolkit), and the atlantoaxial joint equivalent surface is extracted using the moving cube method to build the mesh model of the atlantoaxial joint surface. The mesh model of the atlantoaxial surface is rendered to obtain a flow reconstruction view of the atlantoaxial surface. Analysis of these data provides a more detailed knowledge of the atlantoaxial lateral mass articular surfaces, which can be subsequently used for the optimal design of atlantoaxial fusion devices.

### Data measurement and analysis

**Fig. 3:**
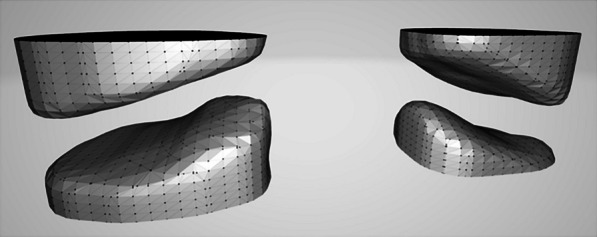
Atlantoaxial articular surface after high-precision gridding (the joint gap has been increased to facilitate observation of the joint surface)

**Fig. 4:**
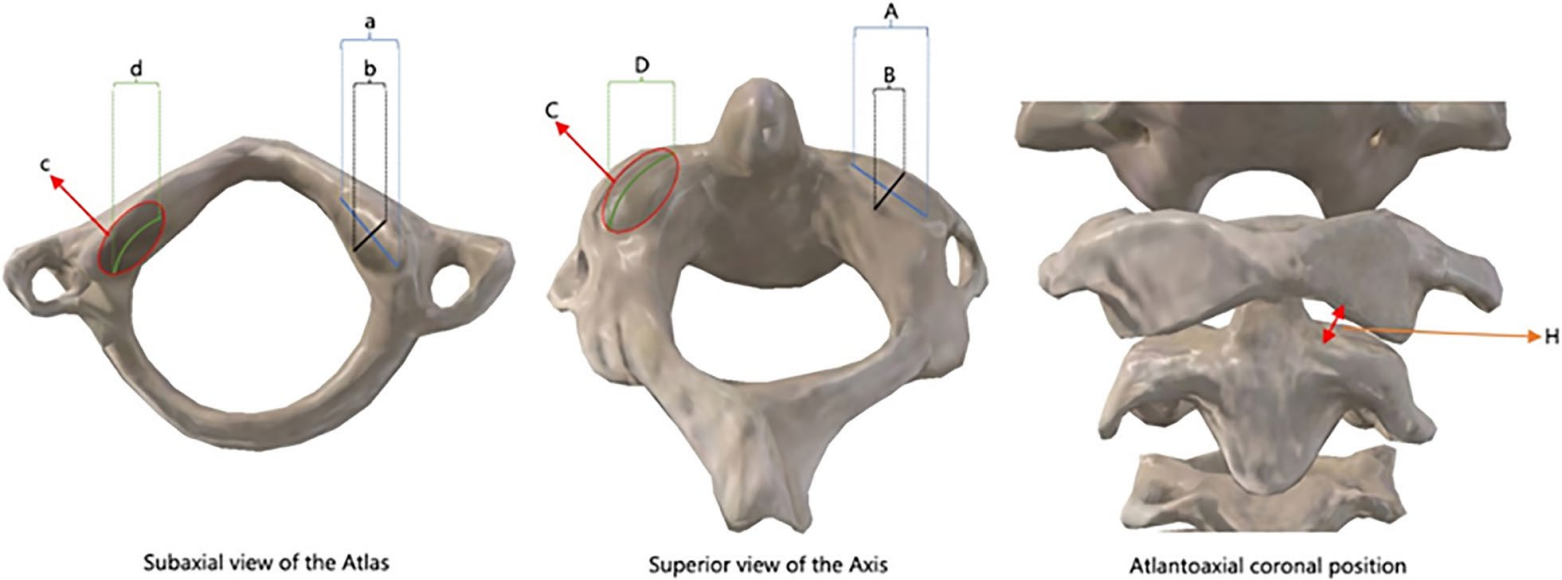
**a** Sagittal diameter of the atlas lateral mass; **b** Transverse diameter of the atlas lateral mass; **c** area of the atlas lateral mass; **d** curvature of the atlas lateral mass; **A** Sagittal diameter of the axis lateral mass; **B** Transverse diameter of the axis lateral mass; **C** area of the axis lateral mass; **D** curvature of the atlas lateral mass; **H** height of the atlantoaxial joint space

**Fig. 5:**
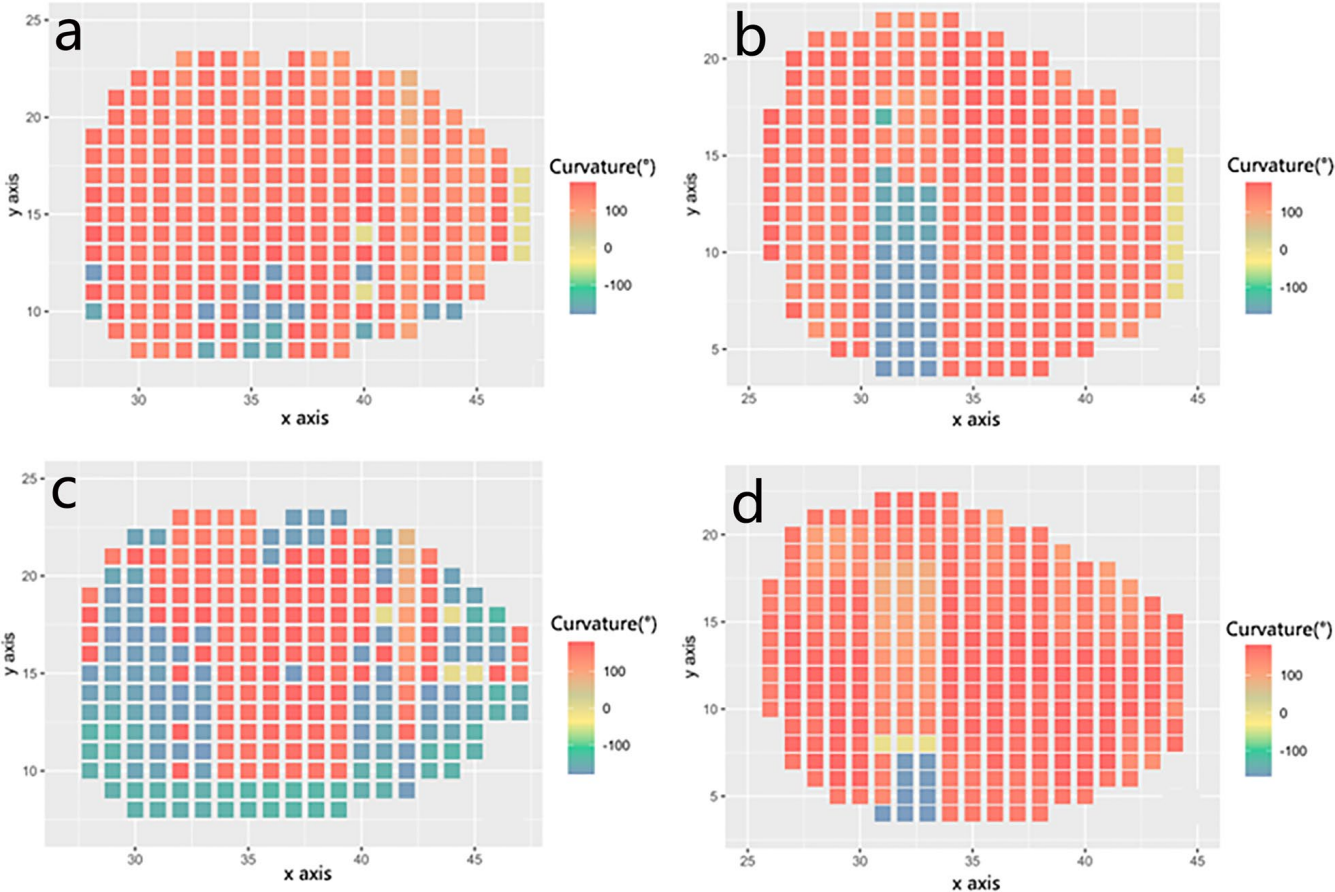
**a** shows the left atlas lateral mass; **b** shows the right atlas lateral mass; **c** shows the left axis lateral mass; **d** shows the right axis lateral mass; x axis: parallel to the coronal plane; y axis: parallel to the sagittal plane

Based on the above atlantoaxial lateral mass articular flow reconstruction model, the articular surface was meshed and divided (Fig. 3). In the high-precision grid, the indices of the left and right sides of the atlantoaxial surface were further measured on each sagittal section by three radiologists (Fig. 4). The inclination angle of the atlantoaxial surface is calculated, and the gap vertical height (perpendicular to the atlantoaxial surface) and the area of the atlantoaxial surface are calculated based on the inclination angle. After the ICC consistency test, the results of the three calculations were averaged. Output of the concavity, curvature, and smoothed atlantoaxial articular surface point cloud model of each point of the articular surface, and plot its distribution on the atlantoaxial articular surface according to the curvature difference of each area (Fig. 5).

The upper and lower articular surfaces were marked as “convex” in the direction of the joint space, “concave” in the opposite direction of the joint space, “superior” in the cephalad side, and “inferior” in the caudal side; in the distribution of atlantoaxial articular surface curvature, the curvature was set as warm color in the upper direction and cool color in the lower direction, and the darker color indicated the greater curvature. The superior articular surface was divided into three equal parts: anterior, middle, and posterior parts of the sagittal diameter, and the inferior articular surface was divided into medial, middle, and lateral parts of the coronal diameter.

### Statistical analysis

SPSS 26.0 statistical software was applied for data analysis, and the measurement data obeying or approximately obeying normal distribution were expressed as the mean ± standard deviation ($\overline{x }$±*s*), and the sagittal diameter, transverse diameter, area and curvature of both sides of the articular surface were tested by paired t test. Measurements from three radiologists were tested for consistency using the ICC consistency test. Differences were considered statistically significant at *P* < 0.05.

## Results

Statistically significant differences between males and females, or between left and right sides of the measurements, were not found in the results of the atlantoaxial lateral mass articular surface index measurements (Tables 2, 3). When the data from the left and right sides were combined, the values were 15.83 ± 1.85 mm sagittal diameter (a), 16.29 ± 2.16 mm transverse diameter (b), 166.53 ± 7.69 mm^2^ articular surface area (c), and 164.03 ± 5.27° articular surface curvature (d) of the atlas lateral mass; 16.22 ± 1.57 mm sagittal diameter (A), 16.49 ± 1.84 mm transverse diameter (B), and 174.48 ± 1.84 mm^2^ articular surface area (C), 153.23 ± 9.03° articular surface curvature for the axis lateral mass (D). The height of the atlantoaxial joint space (H) was 3.05 ± 0.11 mm. The results of the ICC consistency test for the three radiologists showed high consistency, with all ICC > 0.9 (Table 4).

**Table 2:** Atlantoaxial lateral mass articular surface index measurements ($\overline{x }$±s, n = 80)

Indicators	Left	Right	t	P	Bilateral merged
a (mm)	15.28 ± 1.32	15.00 ± 2.38	− 1.571	> 0.05	15.83 ± 1.85
b (mm)	15.65 ± 1.33	15.74 ± 1.32	0.095	> 0.05	16.29 ± 2.16
c (mm^2^)	167.72 ± 5.51	165.34 ± 9.86	− 0.988	> 0.05	166.53 ± 7.69
d (°)	170.35 ± 7.22	171.96 ± 4.95	− 0.367	> 0.05	164.03 ± 5.27
A (mm)	15.69 ± 1.32	16.06 ± 1.71	− 1.503	> 0.05	16.22 ± 1.57
B (mm)	15.55 ± 1.56	15.66 ± 1.30	1.144	> 0.05	16.49 ± 1.84
C (mm^2^)	171.52 ± 6.47	177.43 ± 6.98	0.287	> 0.05	174.48 ± 6.73
D (°)	167.22 ± 5.88	165.52 ± 1.49	0.403	> 0.05	153.23 ± 9.03
H (mm)	3.05 ± 0.13	3.02 ± 0.14	− 0.122	> 0.05	3.05 ± 0.11

**Table 3:** Atlantoaxial lateral mass articular surface vertex distribution (n = 80)

Vertebrae	Side	Coronal position			Sagittal position		
		Internal 1/3	Middle 1/3	External 1/3	Anterior 1/3	Middle 1/3	Posterior 1/3
Atlas	Left	35	40	5	7	65	8
	Right	46	32	2	11	59	10
Axis	Left	2	30	48	9	61	10
	Right	4	32	44	12	57	11

**Table 4:** ICC consistency test results for three radiologists ($\overline{x }$±s, n = 80)

Indicators	A	B	C	ICC (95%CI)
A (mm)	15.54 ± 1.87	15.97 ± 1.92	15.99 ± 1.72	0.986*
b (mm)	15.77 ± 2.22	16.82 ± 1.1.71	16.28 ± 2.36	0.957*
c (mm^2^)	165.39 ± 7.90	167.62 ± 7.80	166.57 ± 7.19	0.988*
d (°)	163.92 ± 5.31	164.37 ± 4.83	163.80 ± 5.63	0.977*
A (mm)	16.34 ± 1.46	15.93 ± 1.55	16.38 ± 1.65	0.983*
B (mm)	16.56 ± 1.70	16.65 ± 2.01	16.25 ± 1.77	0.977*
C (mm^2^)	173.69 ± 7.34	175.43 ± 6.68	174.33 ± 5.99	0.984*
D (°)	154.17 ± 8.53	152.24 ± 9.77	153.28 ± 8.64	0.979*
H (mm)	3.07 ± 0.11	3.04 ± 0.10	3.04 ± 0.12	0.969*

*ICC > 0.9 are considered to be of high consistency

## Discussion

### Current problems with posterior atlantoaxial fusion

Surgical operations in the atlantoaxial region are complex due to the complex bone and neurovascular anatomy of this region, as well as the high levels of joint mobility and the critical physiological functions. CJA have therefore always presented a challenge to spine surgeons. Magerl and Seemann [7] proposed the atlantoaxial transarticular screw internal fixation technique, in combination with the posterior Gallie wiring. The combination of this technique and the posterior Gallie wiring technique provides the best three-dimensional biomechanical stability and the highest fusion rate in posterior atlantoaxial internal fixation fusion. There is excellent resistance to flexion–extension, lateral flexion, and rotation, making it the gold standard procedure of choice for atlantoaxial fusion. On this basis, after continuous innovation and improvement by Goel and Laheri [8] and Harms and Melcher [9], the posterior screw-rod fixation system has become the most widely used posterior atlantoaxial internal fixation technique in clinical practice. It not only has excellent three-dimensional biomechanical stability [10, 11], but it also allows intraoperative repositioning of the atlantoaxial spine by screw-rod translation; it also allows flexible selection of different screw fixation combinations in the atlantoaxial spine according to the preoperative imaging of different patients to achieve the best surgical results. Furthermore, interbody fusion techniques can also be emphasized and used in surgery, but posterior implants cannot be stress-loaded like anterior implants. Posterior instrumentation relies on good contact to achieve bony fusion [12]. In addition, due to the irregular shape and inclination of the lateral atlantoaxial mass articular surfaces [13], the failure of posterior atlantoaxial fusion often occurs when the bone graft material is simply laid between the lateral atlantoaxial mass articular surfaces without stable fixation and compression. Therefore, it is important to accurately measure the data related to the articular surface of the lateral atlantoaxial mass to provide reliable information for the optimization of atlantoaxial fusion devices in the future.

### Measurement of anatomical parameters of the atlantoaxial joint

Methods to study the anatomical and morphological features of the atlantoaxial spine have been developing and advancing, and Lu et al. [14] used calipers and a goniometer to measure bony specimens to determine the length, width, and inclination angle of the upper and lower articular surfaces of the lateral mass joint to provide data to support the placement of anterior transarticular screws. Dong and Rocha et al. [15–17] measured the mean thickness, width, height and coronal inclination of the lateral mass joint using cadavers and found a relatively low incidence of atlantoaxial articular cartilage degeneration. In vitro studies, X-rays were first applied to the anatomic radiological and morphological analysis of the lateral mass joint of the atlantoaxial spine. Xu et al. [18] confirmed the value of indicating the sagittal screw angle of the atlantoaxial spine by lateral X-ray measurements of the lateral mass joint. Ma et al. [19] applied CT and 3D reconstruction to measure the inclination and sliding type of the atlantoaxial lateral mass joint and proposed a staging system for congenital atlantoaxial subluxation to provide a basis for guiding surgical treatment. Other scholars used 3D reconstruction with software such as Mimics and Abaqus based on CT examination and generated finite element models to analyze and measure the range of motion, stress conditions and ligamentous changes of the lateral atlantoaxial mass by changing the model parameters. Accordingly, with the advancement of anatomy, imaging, and multidisciplinary synergy, it has become increasingly complex to study the anatomical morphology of the lateral mass of the atlantoaxial spine based on in vitro radiological images in comparison with anatomical studies on dry skeletal specimens and cadavers. In the future, the study of the anatomical morphology of the atlantoaxial spine will no longer be limited to X-rays, CT and other imaging examinations, but will make use of three-dimensional reconstruction, finite element analysis and other techniques to establish mathematical models and computer simulations of the articular surface of the lateral mass of the atlantoaxial spine. This is so that the morphological, functional and biomechanical studies of the articular surface of the lateral mass of the atlantoaxial spine can be carried out more precisely and accurately.

### Significance of describing the articular surface of the lateral mass of the atlantoaxial spine using differential flow patterns

With the advancement of technology, it is often necessary in modern medicine to use medical imaging data to reconstruct anatomical structures, perform preoperative planning, perform virtual surgery, and simulate clinical surgical operations. It is difficult to form a good fit with the articular surface of the atlantoaxial lateral mass due to the planar end surfaces of all interarticular fusion devices reported in the literature. As a result, the fusion device may not be stable, and the bone graft might not fuse as easily as it should. In this study, the 3D reconstruction of atlantoaxial joint CT images was obtained, and the atlantoaxial articular surface was extracted by python software. The articular surface was fitted into an atlantoaxial surface formed by multiple points, and the “differential manifold” processing method was introduced for the first time to analyze and describe the atlantoaxial lateral mass articulation. The term, “manifold” is not much used in the medical field, but in the past, Zimmer et al. [20] applied manifold embedding to high-dimensional medical image data analysis techniques to automatically select functions and their associated parameters from a pool of candidate functions to generate the best manifold embedding. Xia et al. [21] used low-dose CT to reconstruct manifold networks and achieved good results in terms of visualization and quantification. Maxime et al. [22] characterized the interaction between the heart shape and deformation by nonlinear manifold learning. This high latitude description of heart function is the key to determining heart disease and also confirmed the characteristics of right ventricular disease in the population.

We propose for the first time an implicit surface reconstruction algorithm based on the differential manifold in the processing of atlantoaxial spine image data to measure relevant anatomical parameters on a model with higher accuracy of output. The ROI labeling and measurement portion of this study was completed by three experienced radiologists, and an ICC consistency test of their measurements revealed strong consistency among the three radiologists’ measurements. This means that the algorithm is less affected by human factors and has a certain reliability in clinical extension. Compared with the manual measurement of traditional bony specimens or sagittal and coronal measurements based on image data reconstruction, the error is significantly reduced and the efficiency is significantly improved, which can more accurately characterize the morphology, size, orientation, curvature and other anatomical features of the articular surfaces of the lateral mass of the atlantoaxial spine and provide a deeper understanding of the interaction, motion patterns, stability and function of the articular surfaces of the lateral mass of the atlantoaxial spine. Aside from eliminating the need for expensive commercial software like mimics, this method enables a mathematical model and computer simulation of the atlantoaxial mass’s articular surface that provides tools for further research into its pathological and biomechanical characteristics. By measuring the anatomical morphology of the articular surface of the lateral atlantoaxial mass, this study provides a personalized preoperative assessment for patients undergoing surgery for CJA, provides a database for the individualized design of 3D printed fusion devices, and provides a reference for clinical diagnosis and surgery to reduce surgical risks for the benefit of such patients.

### Measurement and significance of data related to the atlantoaxial articular surface

In previous anatomical studies of the articular surface of the lateral atlantoaxial mass, the sagittal lengths of the articular surface of the atlantoaxial spine measured from cadaveric specimens were (15.7–18.7 mm) and (17.0–17.7 mm), and the coronal lengths were (15.2–16.5 mm) and (16.6–17.3 mm), respectively, and Li et al. [3] measured CT images of 46 adults and obtained The sagittal and transverse diameters of the articular surface of the lateral atlantoaxial mass were 16.96 mm and 16.27 mm, respectively, and the sagittal and transverse diameters of the articular surface of the lateral cardinal mass were 17.69 mm and 16.32 mm, respectively, and similar results were obtained in this study with other anatomical data on the measurement of parameters related to the articular surface of the lateral atlantoaxial mass, which demonstrates that the method used in this study has a high degree of reliability and reliability. The results of this study are similar to the anatomical data of other atlantoaxial mass (Table 5).

**Table 5:** Comparison of measured parameters between previous studies and the present study

Study	Cases	a (mm)	b (mm)	A (mm)	B (mm)	H (mm)	d/D (°)	
Dong et al. [15]	30	15.63 ± 1.04	17.90 ± 1.18					
Rocha et al. [16]	20	18.70 ± 1.60	16.50 ± 2.00					
Kandziora et al. [17]	50			17.00 ± 1.10	16.60 ± 1.25			
Xu et al. [18]	50			17.70 ± 1.30	17.30 ± 1.30			
Li et al. [3]	46	16.96 ± 1.41	16.27 ± 1.36	17.69 ± 1.37	16.32 ± 1.33			
Xiao et al. [23]	60	16.52 ± 1.13	13.66 ± 0.98	14.76 ± 1.06	16.22 ± 1.09	2.99 ± 0.61		
Gu et al. [24]	100	16.38 ± 1.61	16.7 ± 1.61	16.59 ± 1.63	17.14 ± 1.69	3. 39 ± 0.57		171.16 ± 6.21 166.37 ± 8.50
Our study	80	15.83 ± 1.85	16.29 ± 2.16	16.22 ± 1.57	16.49 ± 1.84	3.05 ± 0.11		164.03 ± 5.27 153.23 ± 9.03

In our study, the reconstructed joint gaps were divided into anterior, middle and posterior and internal, middle and external regions by means of differential flow reconstruction surfaces, and it was found that the morphological characteristics of the gaps showed a general trend of anteriorly wide, moderately narrow and posteriorly wide in the sagittal position and internally narrow, moderately wide and externally narrow in the coronal position. This was consistent with the findings of Gu et al. [24]. Meanwhile, the study of the lateral mass of the atlantoaxial spine found that its articular surface curvature has a certain degree of curvature, its projection and depression are different, and there is no exact coincidence or complementary relationship between the two articular bony surfaces. The results of the coronal position with the superior surface vertex generally located in the middle and medial sides were: 93.75% (75/80) and 97.50% (78/80) for the left and right sides of the atlantoaxial spine, respectively, and 97.50% (78/80) and 95.00% (76/80) for the left and right sides of the pivot spine, respectively, with the inferior surface vertex generally located in the middle and lateral sides. In the sagittal position, the results of the vertex of the articular surface in the middle 1/3 were: 81.25% (65/80) and 73.75% (59/80) for the left and right sides of the atlantoaxial spine, respectively, and 76.25% (61/80) and 71.25% (57/80) for the left and right sides of the pivot spine, respectively.

To facilitate the smooth and accurate placement of the fusion device into the lateral atlantoaxial space and to obtain a stable bony fusion, the articular surface of the lateral atlantoaxial mass must be both concave and convex. According to the above measurements, the sagittal apex of the fusion device should be located in the middle 1/3 region, while the coronal apex should be located in the middle and medial regions on the upper surface and in the middle and lateral regions on the lower surface. Combining the results of the above anatomical measurements with the characteristics of the surgical operation during the actual clinical placement of the fusion device, attention should be paid to the design of the anatomical-type fusion device in the next stage with a length of no more than 13 mm, a width of no more than 10 mm, and a fusion device height of 4–6 mm. The overall shape should be that of a folding knife in the sagittal position with the upper surface up-convex and the lower surface up-concave. There should be a date pit shape in the coronal position with the upper surface up-convex in the middle and medial side and the lower surface down-convex in the middle and lateral side.

The sample size for this study was 80 cases, which is relatively small for a detailed anatomical study with individual variation, but is feasible when compared with previous studies due to the complexity of the anatomy at this site which limits the anatomical morphometric studies that can be performed. Previous solid anatomical studies performed on cadavers were limited by the collection of samples, and most current studies involve morphological measurements on digitized images such as CT. The reasons for the small sample size are as follows: First, the sample included in this study was 64-slice cervical spine CT images from a normal population during a physical examination, and not all physical examiners choose cervical spine CT, and even few asymptomatic people choose cervical spine CT to clarify the presence of abnormalities, which led to the limited collection of the sample size in this study. Second, the anatomy of atlantoaxial joint is complicated, which makes it difficult to carry out the study of anatomical morphometry in this region; third, the surface reconstruction algorithm based on differential manifolds needs to go through a series of complicated operations, such as ROI labeling, atlantoaxial surface extraction, and equivalent surface extraction, which are difficult and lengthy to process, again resulting in small sample sizes. Also, the age distribution of the sample included in this study was 20–54 years old, with a mean age of 37.8 years, which are mostly younger populations with less joint degeneration and hyperplasia, which could avoid the influence of joints with osteophytes due to aging in the older population on this measurement. However, sampling error due to small sample size is unavoidable, and larger sample sizes need to be further included to conduct the study, enabling the results of this study to be generalized in clinical settings.

## Limitations

(i) For the results to be verified, the sample size included in the study is somewhat small, and further expansion of the sample size is required; (ii) Unlike the direct measurement of specimens, this study is an imaging anatomy study based on the reconstructed images of CT scans, and there may be a certain degree of error in the selection of thresholds and fitting of joint surfaces to the real object; (iii) Due to the selection and setting of the algorithm, there may be some errors in controlling grid accuracy and curvature determination.

## Future research directions

It is necessary to further explore the optimization of data algorithms and to study the data related to the articular surface of the lateral mass of the atlantoaxial spine in patients with CJA by fitting models of the articular surface of the lateral mass of the atlantoaxial spine in conjunction with computer and other engineering, information engineering, biomechanics and other specialties. We will use the data from this study to further optimize the lateral atlantoaxial mass fusion device design and verify its implantability and safety, laying the foundation for the next animal experiments and clinical trials.

## Conclusion

1. Clinical imaging data and differential flow algorithms can be used to better visualize the anatomical structure of the articular surfaces of the lateral atlantoaxial mass, measure all of the relevant parameters accurately, and provide data for the design of anatomical lateral atlantoaxial mass joint fusions.
2. The anatomical shape of the articular surface of the lateral atlantoaxial mass is irregular, with a certain curvature of the articular surface and different positions of the raised and depressed articular surfaces. The two bony surfaces do not show a strict coincidence and complementary relationship, and the positions of the upper and lower convexity can show a certain pattern in the population in general.
3. The atlantoaxial articular surface of most healthy adults is morphologically adequate to accept fusion devices up to 13 mm in length, 10 mm in width, and 4–6 mm in height. Based on the measured curvature pattern of the articular surface, anatomic fusion devices are generally shaped like a folding knife in the sagittal position and are more likely to fit the articular surface in the coronal position with a date palm shape.
4. There is a wide variation in the anatomical morphology of the lateral mass of the atlantoaxial spine which is large, and a thorough preoperative imaging examination of the atlantoaxial spine should be performed to provide a comprehensive preoperative evaluation of the patient, so that a reasonable surgical plan can be formulated and individualized treatment can be achieved.

## Data Availability

All data generated or analyzed during this study are included in this published article. The datasets used and/or analyzed during the current study are available from the corresponding author on reasonable request.

## Abbreviations

CJA: Craniocervical junction abnormalities
CT: Computer tomography
MRI: Magnetic resonance imaging
DICOM: Digital imaging and communications in medicine
PACS: Picture archiving and communication system
ITK: Insight toolkit
ROI: Region of interest
VTK: Visualization toolkit

## References

[1] Goel A (2018). Craniovertebral junction instability-an overview. World Neurosurg.

[2] Salunke P, Karthigeyan M, Kodigudla MK, Kelkar AV, Goel VK (2022). C1–C2 arthroplasty for craniovertebral junction instability: a preliminary proof of concept in human cadavers. J Craniovert Junct Spine.

[3] Li S, Ni B, Xie N, Wang M, Guo X, Zhang F, Wang J, Zhao W (2010). Biomechanical evaluation of an atlantoaxial lateral mass fusion cage with C1–C2 pedicle fixation. Spine.

[4] Park J, Scheer JK, Lim TJ, Deviren V, Ames CP (2011). Biomechanical analysis of Goel technique for C1–2 fusion. J Neurosurg Spine.

[5] Tan M, Wang H, Wang Y, Zhang G, Yi P, Li Z, Wei H, Yang F (2003). Morphometric evaluation of screw fixation in atlas via posterior arch and lateral mass. Spine.

[6] Yan S, Xu D, Zhang B, Zhang HJ, Yang Q, Lin S (2007). Graph embedding and extensions: a general framework for dimensionality reduction. IEEE Trans Pattern Anal Mach Intell.

[7] Renna R, Plantone F, Plantone D (2013). Atlantoaxial subluxation in rheumatoid arthritis. J Rheumatol.

[8] Goel A, Bhatjiwale M, Desai K (1998). Basilar invagination: a study based on 190 surgically treated patients. J Neurosurg.

[9] Schulz R, Macchiavello N, Fernández E, Carredano X, Garrido O, Diaz J, Melcher RP (2011). Harms C1–C2 instrumentation technique: anatomo-surgical guide. Spine.

[10] Yang SY, Boniello AJ, Poorman CE, Chang AL, Wang S, Passias PG (2014). A review of the diagnosis and treatment of atlantoaxial dislocations. Global Spine J.

[11] Ma H, Dong L, Liu C, Yi P, Yang F, Tang X, Tan M (2016). Modified technique of transoral release in one-stage anterior release and posterior reduction for irreducible atlantoaxial dislocation. J Orthop Sci.

[12] Singh DK, Shankar D, Singh N, Singh RK, Chand VK (2022). C2 Screw fixation techniques in atlantoaxial instability: a technical review. J Craniovert Junct Spine.

[13] Goel A (2022). Artificial atlantoaxial and subaxial facetal joint—proposal of models. J Craniovert Junct Spine.

[14] Sonone S, Dahapute AA, Waghchoure C, Marathe N, Keny SA, Singh K, Gala R (2019). Anatomic considerations of anterior transarticular screw fixation for atlantoaxial instability. Asian Spine J.

[15] Dong Y, Hong MX, Jianyi L, Lin MY (2003). Quantitative anatomy of the lateral mass of the atlas. Spine.

[16] König SA, Goldammer A, Vitzthum HE (2005). Anatomical data on the craniocervical junction and their correlation with degenerative changes in 30 cadaveric specimens. J Neurosurg Spine.

[17] Christensen DM, Eastlack RK, Lynch JJ, Yaszemski MJ, Currier BL (2007). C1 anatomy and dimensions relative to lateral mass screw placement. Spine.

[18] Xu R, Ebraheim NA, Misson JR, Yeasting RA (1998). The reliability of the lateral radiograph in determination of the optimal transarticular C1–C2 screw length. Spine.

[19] Ma F, He H, Liao Y, Tang Q, Tang C, Yang S, Wang Q, Zhong D (2020). Classification of the facets of lateral atlantoaxial joints in patients with congenital atlantoaxial dislocation. Eur Spine J.

[20] Zimmer VA, Lekadir K, Hoogendoorn C, Frangi AF, Piella G (2015). A framework for optimal kernel-based manifold embedding of medical image data. Comput Med Imaging Graphics.

[21] Xia W, Lu Z, Huang Y, Shi Z, Liu Y, Chen H, Chen Y, Zhou J, Zhang Y (2021). MAGIC: manifold and graph integrative convolutional network for low-dose CT reconstruction. IEEE Trans Med Imaging.

[22] Maxime DF, Pamela M, Patrick C, Nicolas D (2022). Characterizing interactions between cardiac shape and deformation by non-linear manifold learning. Med Image Anal.

[23] Xiao H, Luo MW, Xie SW (2020). Design an atlantoaxial lateral mass fusion cage based on CT measurement. J Gannan Med Univ.

[24] Gu YJ, Zhang JH, Yu L (2021). Radiological study on the anatomical morphology of atlantoaxial lateral mass articular surface. Chin J Anat Clin.

